# Plasmonic Biosensors for Single-Molecule Biomedical Analysis

**DOI:** 10.3390/bios11040123

**Published:** 2021-04-15

**Authors:** Elba Mauriz, Laura M. Lechuga

**Affiliations:** 1 Department of Nursing and Physiotherapy, Universidad de León, Campus de Vegazana, s/n, 24071 León, Spain; 2 Institute of Food Science and Technology (ICTAL), La Serna 58, 24007 León, Spain; 3 Nanobiosensors and Bioanalytical Applications Group, Catalan Institute of Nanoscience and Nanotechnology (ICN2), CSIC, BIST, and CIBER-BBN, Campus UAB, 08193 Barcelona, Spain; laura.lechuga@icn2.cat

**Keywords:** single-molecule analysis, biosensors, plasmonics, nanoparticle, nanostructure, nucleic acids, virus, living-cells

## Abstract

The rapid spread of epidemic diseases (i.e., coronavirus disease 2019 (COVID-19)) has contributed to focus global attention on the diagnosis of medical conditions by ultrasensitive detection methods. To overcome this challenge, increasing efforts have been driven towards the development of single-molecule analytical platforms. In this context, recent progress in plasmonic biosensing has enabled the design of novel detection strategies capable of targeting individual molecules while evaluating their binding affinity and biological interactions. This review compiles the latest advances in plasmonic technologies for monitoring clinically relevant biomarkers at the single-molecule level. Functional applications are discussed according to plasmonic sensing modes based on either nanoapertures or nanoparticle approaches. A special focus was devoted to new analytical developments involving a wide variety of analytes (e.g., proteins, living cells, nucleic acids and viruses). The utility of plasmonic-based single-molecule analysis for personalized medicine, considering technological limitations and future prospects, is also overviewed.

## 1. Introduction

**Figure 1:**
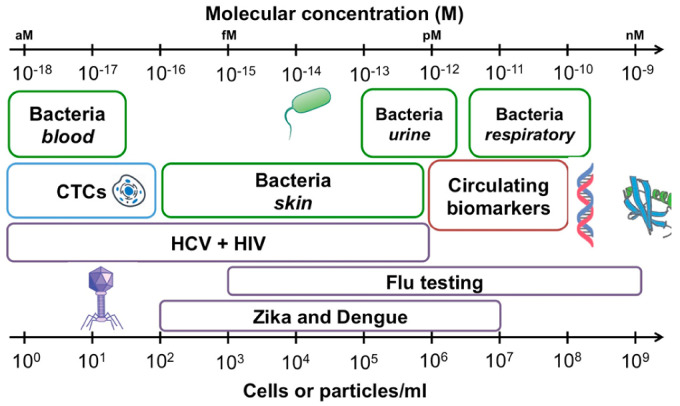
Concentrations of common analytes in biological samples expressed as cells or particles per milliliter of sample (bottom axis), and molarity (top axis). Bacterial concentrations are outlined in green, viral targets are outlined in purple, circulating tumor cells (CTCs) are shown in blue and circulating biomarkers including proteins and nucleic acids in red. Reprinted with permission from Kelley et al. [2] Copyright © 2017 American Chemical Society.

The detection of clinical biomarkers with relevant sensitivity is becoming a great concern for biomedical analysis [1,2,3]. The objective quantification of biological targets (i.e., nucleic acids, proteins, cells and microorganisms) enables both the identification of normal or pathological diagnostic outcomes and the monitoring of the biological response to therapeutic drugs or environmental agents. Typically, chemical and biological species are present in clinical samples at trace concentrations as low as attomolar levels [1,2,4]. Measuring such small amounts of biomolecules is particularly relevant not only for the early identification of cancer biomarkers but also for the rapid recognition of pathogenic agents and the prevention of potential outbreaks (Figure 1) [2,5]. For instance, the precise diagnosis of bacterial and virus infections such as tuberculosis, hepatitis, Acquired Immune Deficiency Syndrome (AIDS) and more recently coronavirus disease 2019 (COVID-19) may require the transduction of independent events occurring at single-molecule or cellular levels [2,4]. Single-molecule analysis is also needed when only a minimum volume of sample is available or multiplexed analysis is demanded to target various analytes simultaneously in a short time [3,6].

Meeting these challenges involves the monitoring of single-molecule interactions between reacting species via sensing devices that can distinguish binding kinetics in heterogeneous samples with sufficient specificity [1,4]. The observation of a signal from an individual molecule within the ensemble also relies on the transport of the analytes to the sensing region. Therefore, strategies to improve single molecule detection should take into consideration both the sensing mechanism and the diffusion rate, analyte concentration and sample volume [6].

Current research in single-molecule bioanalytical technologies has been primarily focused on configurations based on total internal reflection fluorescence (TIRF) and fluorescent-related methods [1,7,8]. Other optical detection techniques such as surface-enhanced Raman scattering (SERS), total internal reflection scattering (TIRS), dark-field microscopy (DFM), or near-field scanning optical microscopy (NSOM) have also demonstrated their value for the determination of single-molecule interactions [9,10].

From this perspective, plasmonic-based techniques take advantage of the unique optical and electronic properties of plasmonic nanostructures to assess the signals and kinetic distributions of single-molecule binding events occurring in real-time [11,12]. Nanoplasmonic sensing principles rely on the collective oscillation of free electrons known as localized surface plasmons at metallic interfaces or within nanostructures under light stimulation [13,14,15]. The possibility of providing sensitive and selective analysis using single-molecule sensing mechanisms depend on the signal amplification resulting from the enhancement of the intensity of the optical field [8]. Therefore, the manipulation of the physicochemical environment and composition of plasmonic nanostructures allow the precise confinement of the electromagnetic field into nanoscale volumes of only a few nanometers, which are named “hot spots”. The generation of extreme electric field gradients facilitates optical trapping of single molecules while providing longer dwelling times [16,17]. Particularly, the interaction between a biological receptor and its target analyte near the optical-field can be detected through the shift of the plasmon polariton resonance wavelength induced by a local refractive index change [13].

**Figure 2:**
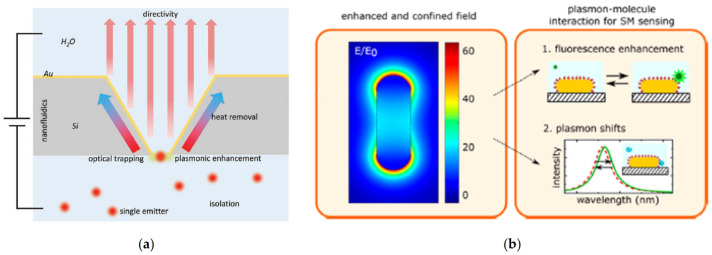
Schematic representation of plasmon-enhanced single-molecule sensing using nanoapertures and nanoparticles: (**a**) Nanoapertures (rectangle in an etched silicon film) in metal films (gold) for enhanced interaction with single emitters (shown as red glowing circles). The anisotropic etch produces a horn antenna for directive coupling. The aperture provides plasmonic enhancement for optical trapping. The aperture also serves as a nanopore for nanofluidic functionality, like monitoring ionic current from flow through the aperture Adapted with permission from Gordon et al. [27] Copyright © 2020 Wiley; (**b**) Nanoparticles: The plasmon resonance induces a strongly enhanced and tightly confined local field around the particles. The field shown here is for a gold nanorod that is excited on resonance. The local field mediates plasmon−molecule interactions, enabling enhanced single-molecule detection by monitoring plasmon-induced changes of the molecule (resulting in, e.g., fluorescence enhancement) or by monitoring molecule-induced changes of the plasmon (resulting in frequency-shifts of the plasmon). Adapted with permission from Taylor et al. [4] Copyright © 2017 American Chemical Society.

The design of high-spatial-resolution surface structures is very valuable for the surface-enhanced sensing of individual events (Figure 2) [18]. In this sense, plasmonic single-molecule approaches mainly rely upon the extinction spectra of metallic nanoparticles (NPs) and the capacity of optical nanoapertures (i.e., arrays of nanoholes, nanopores, nanowells) to confine the electromagnetic field into ultrasmall subwavelength dimensions [1,8,9,19,20,21,22,23]. Both types of plasmonic nanostructures have been successfully applied to the label-free quantification of single-proteins in the diagnosis of acute and chronic diseases in early stages, the recognition of single-nucleotide specificity and the tracking of independent biological processes in living cells inserted in nanopores [24,25,26].

Although the fundamentals and functionalities of single-molecule biosensing have been extensively addressed in previous reviews [1,8,22], the specific role of plasmonic biosensors in the detection of single-molecule biological targets has still not been satisfactorily resolved. Therefore, the aim of this work is to summarize recent progress in plasmonic single-molecule biosensing schemes for biomedical applications. This review particularly concentrates on the quantification of clinically relevant biomarkers using plasmonic nanostructures such as metallic nanoparticles and optical nanoapertures. Recent research in the design of the new generation of plasmonic platforms for practical sensing of independent molecular interactions from the perspective of point of care testing is also reviewed.

## 2. Plasmonic Nanoapertures for Single-Molecule Analysis 

The exploitation of plasmonic nanoapertures as sensing probes for single-molecule analysis has attracted great interest over the last decade. The formation of nanoapertures in an opaque metal film permits the confinement of light in the subwavelength regime, thereby limiting the interaction volume to the nanometer scale [28,29]. 

**Figure 3:**
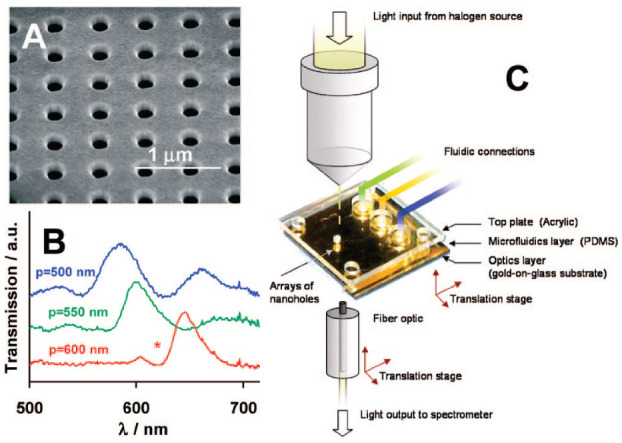
(**A**) Scanning electron micrograph (SEM) of an array of nanoholes in a gold film; (**B**) Extraordinary Optical Transmission (EOT) spectra for three arrays with different periodicities; (**C**) experimental setup to measure the EOT effect. The metal film is deposited on a glass slide, and the gold side of the array is exposed to solvents and aqueous solutions delivered by microfluidics. Adapted with permission from Gordon et al. [19] Copyright © 2008 American Chemical Society.

Among nanoscale apertures, solid-state nanopores provide the basis for the development of stable and durable sensing platforms capable of achieving extremely low single-molecule detection limits with high specificity [1,30]. Nanopore sensors consist of an electrically thin insulating membrane traversed by 1–100 nm pores through which ions can cross [22,31]. When charged molecules pass through the pore, the application of a voltage between two electrodes produces the interaction with the membrane and the displacement of ions, causing the change of the ionic current. This displacement can be determined via optical or electrical sensing [1,22]. The optical sensing mainly depends on the change of the refractive index after the arrival of the target molecule to the hotspot of the plasmonic nanopore, wherein the electromagnetic field is strongly localized (Figure 3) [19]. Specifically, the optical transmission through the nanoaperture conducts the plasmon resonance to the far field in which the individual molecule can be detected. Monitoring of the plasmon resonance redshift can either be performed by registering the plasmon resonance peak or by tracking the scattered light intensity at a fixed excitation wavelength. In this way, plasmonic nanoapertures enable the ultrasensitive quantification of molecular interactions by combining the acquisition of bimodal optical and electrical data [32].

Since nanopore sensors facilitate the active transport of charged molecules at very low concentrations towards the nanopore aperture from long distances, the combination with plasmonic sensing can contribute to improve the diffusion of single-molecules at a plasmonic hotspot. In addition, the nanoscale diameter of the nanopore is comparable to the cross-section of the molecule, thus reducing the molecule motion to only one dimension. This feature allows both the increment of the dwelling time and the enhancement of the signal-to-background ratio [22]. Therefore, by controlling the nanopore diameter, analytes can be confined to a restricted space and the capture time increases significantly. Accordingly, the reduction in the nanopore size contributes to enhancing the signal-to-noise ratio results enabling highly selective single-molecule detection.

These interesting properties have paved the way for a variety of biological applications aimed at manipulating nanoscale molecules such as single-proteins, virus like-particles or nucleic acids. A detailed description of the fabrication of nanopores has been comprehensively reviewed in previous works and it is beyond the scope of this review [22,33]. Novel developments in plasmonic nanopore sensing schemes for evaluating biological processes including protein-protein interactions, translocation of single proteins, assessment of kinetic reactions, analysis of RNA structure, single nucleotide polymorphisms and DNA sequencing approaches are shown in this section (see Table 1).

### 2.1. Single-Nucleic Acid Nanopore Detection

Nucleic acid analysis is vital for the diagnosis of diseases such as cancer and infectious conditions. Recent technological advancements have permitted the emergence of DNA sequencing as a viable approach to obtain specific information about the disease onset and progress. Specifically, the recognition of single nucleotides, adenine (A), thymine (T), cytosine (C), and guanine (G), is the primary aim of the synthesis-sequencing strategies [26,34,35,45,46]. In this vein, nanopore biosensors have proved to be compatible with the determination of DNA nucleotides at the single-molecule level with sufficient sensitivity and accuracy. Particularly, the sequence of DNA bases can be determined by monitoring current changes when the different DNA bases pass through the nanopore. Although DNA sequencing has been extensively investigated through nanopore detection strategies, several important challenges still remain unresolved.

For example, single molecule analysis of nucleic acids using nanopores present various limitations due to the low control of certain parameters such as high salt conditions, high temperature and pH [34,35,36]. Likewise, the limited residence time of DNA nucleotides inside the nanopore and the elevated ionic current noise are also potential drawbacks of nanopore sensors [45]. The integration of an optical readout into nanopore structures offers potential benefits for the specific detection of DNA bases.

A simulation approach was used to improve the detection efficiency of double-stranded single DNA molecules by means of subwavelength plasmonic nanopore-nanowell devices with fluorescence enhancement [34]. The most important benefits of the method rely upon the ability to synchronize the optical and electrical signals while providing the suppression of the fluorescence background and the enhancement of the fluorescence signal. This study demonstrated that the signal was stronger and the background smaller in several orders of magnitude due to the abolition of the stochastic activity associated with the DNA motion near the nanopore and the enhancement in the observed fluorescence intensity.

**Figure 4:**
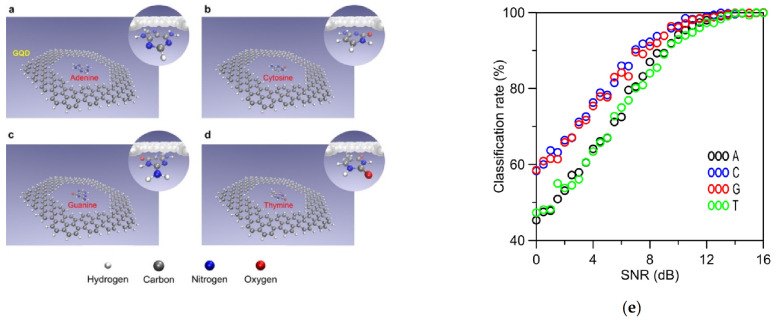
Graphene Quantum Dots (GQD) pores with nucleobases of (**a**) adenine, (**b**) cytosine, (**c**) guanine, and (**d**) thymine. For all the cases from (**a**–**d**), the length of the hexagonal GQD side was A = 1.8 nm and the side length of the pore was D = 1.0 nm. All the structures are relaxed and also binding atoms (hydrogen) to the pore and GQD edges are included. (**e**) Classification rates of unknown DNA nucleobases inserted into GQD (1.8,1.0) to nucleobases as a function of the signal-to-noise ratio (SNR). The classification rate is the percentage of the noisy samples, which are classified correctly to a type of DNA nucleobase. Adapted with permission from Abasifard et al. [35] Copyright © 2019 American Chemical Society.

Another singular strategy made use of experimental calculations to study the effects of the optical absorption spectrum of DNA bases on the interband plasmons of graphene quantum dots [35]. The work also investigated the sensitivity and selectivity factors as well as the association between the insertion of DNA bases and the optical excitation of graphene quantum dots within nanopores (Figure 4). The results indicated that among the four hexagonal graphene quantum dots’ structures examined, inserted DNA molecules provided higher shift increments in the energy of the peaks for structures with a pore side length of 1.0 nm. Similarly, the effect of DNA-graphene interactions and nucleobase rotation was studied in order to explore the applicability of the proposed method for DNA sequencing. Specifically, all the DNA nucleobases were inserted into the pore in a rotated mode, yielding almost no effect on the energy of the peaks for G and T, in the case of 90° rotation. Regarding A and C, most of the peaks were affected by the rotation of the nucleobase and could be easily differentiated from the peak shifts of the other nucleobases in both rotated and nonrotated modes. Finally, the noise level was investigated in order to classify any unknown inserted nucleobase into one of the possible types A, C, G, and T. A true classification rate greater than 95% was obtained when the signal to-noise ratio was higher than 12 dB (Figure 4). Therefore, the proposed method suggests that high-speed plasmonic signals and forces can overcome current limitations of solid-state nanopores and two-dimensional materials such as fast-DNA translocation, slow sensing mechanisms and increased noise levels.

**Figure 5:**
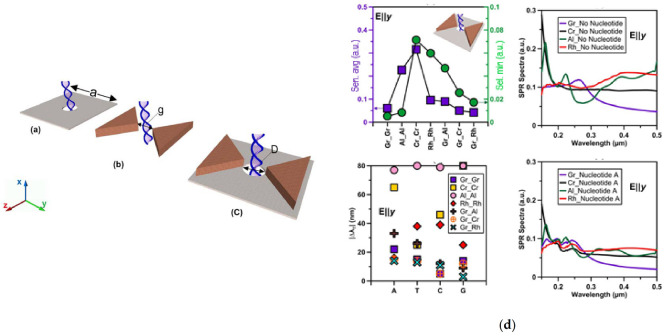
(**a**) Schematic illustration of the proposed structures and the DNA molecule present. (**a**) Nanosheet with a pore (nanopore structure); (**b**) Bowtie structure. (**c**) Bowtie-nanopore compound structure. (**d**) Bowtie-nanopore combination, a = 3 nm, D = 2 nm, g = 1.5 nm and the bowtie side length was 2 nm. Graph (upper left) shows minimum selectivity and average sensitivity. Graph (down left) shows total peak wavelength shifts relative to the condition where no nucleotide was present. Graph (Upper right) shows SPR spectra with no nucleotide. Graph (down left) shows SPR spectra in the presence of nucleotide A. Adapted with permission from Fotouhi et al. [36] Copyright © 2016 Optical Society of America.

The application of plasmonics to the sensing of individual DNA molecules can be particularly promising for DNA sequencing. An interesting strategy investigated the effect of different materials like chromium (Cr), aluminum (Al), rhodium (Rh), and graphene (Gr) on the changes of the surface plasmon resonance (SPR) spectra in the UV region of light when single nucleotides pass through: nanopore, bowtie, and bowtie-nanopore compound structures (Figure 5) [36]. The work focused on the optical characterization of the proposed structures with regard to the plasmonic properties of distinct materials using a discrete dipole approximation method. However, the sensitivity and selectivity factors were also calculated taking into account the dimension of the nanostructures. The nanopore structure, which showed higher sensitivity to nucleotides was the Gr-based bowtie, whereas Cr-based compound structures displayed better selectivity. Therefore, when increasing the pore diameter or gap distance, both sensitivity and selectivity factors decrease. These results indicate that the presentation of DNA nucleotides through bowtie-nanopore compound structures could generate more shifts in the plasmonic spectra than the nanopore or bowtie structures. This is the crucial aspect for DNA sequencing, since the selectivity factor enables the differentiation between all the four possible nucleotides from a single-stranded DNA. In contrast, the sensitivity factor is more important for DNA sensing or translocation detection. Consequently, the plasmonic properties of nanostructures contribute to reduce DNA translocation speed while permitting higher ionic or tunneling signals in the pore in comparison to nanopore DNA sequencers, thus laying the basis for practical DNA sequencing.

**Figure 6:**
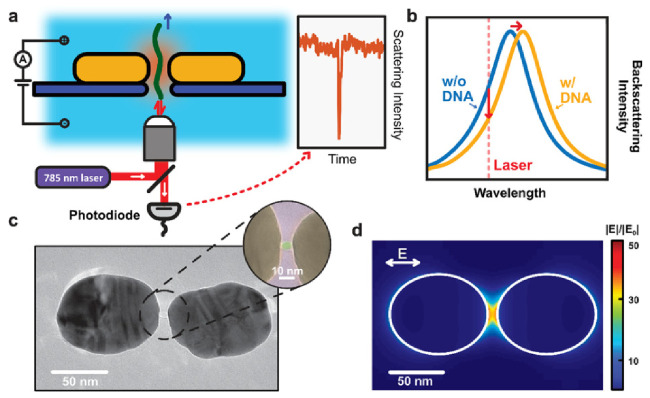
Plasmonic nanopores for single-molecule optical sensing. (**a**) Schematic side-view illustration of a DNA molecule that is electrophoretically driven through a plasmonic nanopore and detected by optical backscattering from the plasmonic antenna. (**b**) Illustration of the sensing principle. The temporary presence of the DNA in the hotspot region of the plasmonic antenna induces a shift of the resonance wavelength of the antenna, hence decreasing the scattering intensity that is detected at the excitation laser wavelength. (**c**) Transmission electron microscopy (TEM) image of the plasmonic nanopore devices used in the experiments. The plasmonic nanopore consists of a gold dimer antenna with a ~5 nm nanopore at the gap center. The inset shows a false colored TEM image of a zoom of the nanogap region, highlighting the nanopore. (**d**) Simulated electromagnetic field distribution of the plasmonic nanopore in longitudinal excitation with a wavelength of 785 nm. The simulation shows the extremely enhanced and confined electromagnetic field within the gap of the dimer antenna, which is required for label-free optical sensing of single molecules. Reprinted with permission from Shi et al. [37] Copyright © 2018 American Chemical Society.

Another plasmonic nanopore sensor demonstrated the optical detection of single DNA molecules at high acquisition rates by monitoring the backscattered light intensity from the plasmonic nanoaperture [37]. This approach took advantage of both the electrophoretic properties of the nanopore to attract molecules through it and the enhanced electromagnetic field hotspots of the nanoantenna (Figure 6). The translocation of individual DNA molecules in the nanopore gap was monitored due to the changes of the intensity of light backscattered from the nanoantenna. The principal benefit of the proposed method was the precision for delivering DNA molecules into plasmonic hotspots through a solid-state nanopore. The structure of the plasmonic nanopore, consisting of two elongated nanodiscs in a gold bowtie nanoantenna, also allows the efficient reuse of the sensor by driving biomolecules away from the hotspot after signal acquisition. Likewise, the ability to reduce the waiting time to sub-milliseconds while improving the time resolution and maximum number of events in several orders of magnitude are additional advantages over previously reported plasmon resonance-based single-molecule sensing methods [47,48,49,50]. Further advancement of the method in terms of sensitivity and selectivity could be achieved by improving the nanoantenna design and the biochemical modification of the gold surface.

The translocation of single DNA molecules is described by Dekker’s group [38] using an inverted-bowtie gold plasmonic nanopore of 100 nm. In this work, the sensing probe examined DNA−protein interactions at physiological conditions. In particular, DNA translocation was evaluated through the characterization of optical signals under different illumination conditions, showing no significant association with driving voltages and electrolyte concentrations. Although the proposed method demonstrated that the optical transmission was induced by translocating DNA molecules, the improvement of the detection scheme was also suggested in order to enhance the plasmon resonance shift when the DNA molecule was present in the gap of the nanoantenna.

### 2.2. Single-Protein Nanopore Detection

Solid-state nanopores sensors have been utilized in the monitoring of single-protein interactions by a significant number of developments [51,52,53]. Conversely, the integration of plasmonic nanoapertures and solid-state nanopores still has limited applications in the transcriptomics field for the determination of protein size, charge and structure.

**Figure 7:**
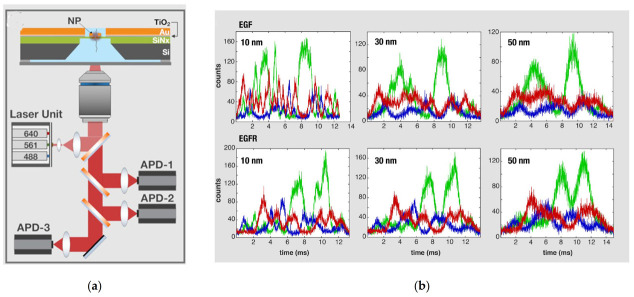
(**a**) Schematics of the nanopore chip and optical system, which includes a high numerical apertures water immersion objective lens, three excitation laser lines and corresponding Silicon avalanche photodiodes (APDs). The nanopore chip is made of four consecutive layers: silicon (grey), silicon nitride (green) in which the nanopore is drilled, titanium oxide (grey blue) and gold (orange); (**b**) optical signals simulated using three distinct spatial resolutions: 10, 30 and 50 nm (from left to right). Adapted with permission from Ohayon et al. [40] Copyright © 2019 PLOS.

Recent research in combining plasmonic nanopore sensing is transforming the proteomics field and a few works have developed singular detection methods to discriminate proteins at the molecular level. In this sense, Ohayon et al. reported a plasmonic-nanopore biosensor that made use of a simulation approach based on tri-color fluorescence and pattern-recognition algorithms for the identification of nearly 10^8^ individual protein translocations [40]. This method was applied to the recognition of human plasma proteome and commercial cytokine panels with an accuracy of ~98%. The model first evaluated the suitability of the plasmonic structure on the top of the nanopore by using Finite Difference Domain (FDTD) computations. Secondly, partial fluorophore labelling of each of the three target amino acids (lysine, cysteine and methionine) was achieved applying the simulation to each protein. Lastly, negatively charged SDS (sodium dodecyl sulfate polyacrylamide)-denatured proteins were illuminated at three wavelengths when passing through the plasmonic nanopore (sub-5 nanometer). This passage allowed the generation of optical fingerprints (three-color fluorescence time traces) and the subsequent analysis with a deep-learning algorithm (Figure 7). The proposed method suggests potential advantages over current detection techniques in terms of accuracy and robustness. Additionally, the versatility of this strategy allows the identification of the whole proteome without depending exclusively on the ion-current measurements through the pore, thus leading to the parallel readout provided by multi-pixel single-photon sensors.

Another interesting approach described a plasmonic nanopore biosensor that took advantage of the combined effect of the electrophoretic and the optical trapping force from the nanopore and the nanoaperture, respectively. In particular, this work utilized an inverted-bowtie plasmonic nanopore to retain individual beta-amylase proteins [41]. The plasmonic nanoaperture shaped in a 100 nm/20 nm thick gold/silicon-nitride film allowed the electrical distribution of molecules to the nanopore while ensuring the nano-optical trapping of single-proteins and the collection of the enhanced optical transmission signal as a readout. The capacity of the plasmonic nanopore to act as nano tweezer was first demonstrated by trapping 20 nm polystyrene (PS) nanoparticles inside the nanopore. On the other hand, the optical trapping of beta-amylase protein, a globular 200 kDa protein of around 10 nm in size, in the plasmonic nanopore yielded larger residence times and significant surface interactions. The detection signal of the protein was also characterized by applying a transmembrane bias voltage that resulted in the increase in the event rate over an order of magnitude and the decrease in the residence times. Hence, the combination of the optical trapping and the electrophoretic forces contributed to enlarge the control over single molecules and their motion toward the sensor. Finally, the investigation on the effect of protein-surface interactions demonstrated the absence of consistent trapping for larger proteins in comparison to beta amylase, thus indicating that the optical forces need to be strengthened in the absence of surface interactions. Therefore, although this method provided external control at the single-molecule level for proteins below 200 kDa, future work is needed to ascertain the contribution of nano tweezing for a wider variety of different biomolecules.

### 2.3. Single-Molecule Detection of Pathogenic Agents

Infectious diseases are a major cause of global concern in the present day due to their persistence and rapid transmission among populations [5,54]. Their enormous impact on the global economy and public health result in a prevalence of over 3.1 billion cases and a mortality rate of ~15% worldwide (8.2 million) in 2016 [54] Therefore, the need for efficient diagnostic strategies has dramatically increased owing to the health risks posed by recent disease outbreaks, and the threat of global pandemics such as that caused by COVID-19. In this context, ultrasensitive quantification of pathogenic microorganisms via high throughput analytical methods at the single-molecule level is strongly needed. The development of plasmonic nanopore technologies has permitted the detection of various infectious agents including viruses and bacteria.

**Figure 8:**
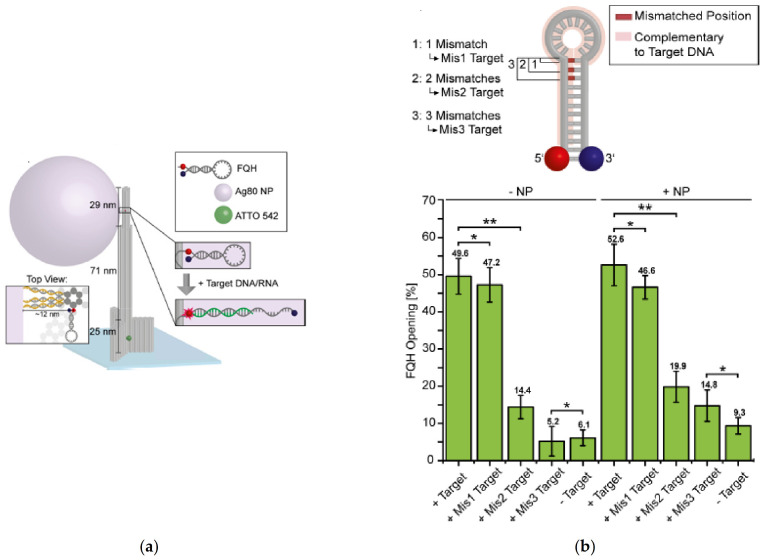
(**a**) DNA origami pillar with a total height of 125 nm is immobilized via biotin modifications on a BSA-biotin/neutravidin surface in a low concentration to ensure single molecule detection. An ATTO 542 dye is incorporated into its bottom part to localize the origami (green dot in sketch). On its upper part, a fluorescence-quenching hairpin (FQH) is bound to the pillar by extending its strand on the 5′ end. This allows incorporation of the FQH into the origami pillar during folding of the nanostructures (insets). Upon the addition of specific target DNA/RNA, the FQH is opened resulting in red fluorescence. To enhance the fluorescence arising from the FQH a silver nanoparticle (NP, 80 nm diameter) is attached to the origami as shown in the top view. The thiol-labeled oligonucleotides hybridize with the protruding strands of the DNA pillar; (**b**) Influence of single-nucleotide variations on hairpin opening, showing the schematic representation of mismatched positions of the target DNA complementary to the FQH sequence. Mis1 target has one mismatch located at position 1, Mis2 target two mismatches at position 2, and Mis3 target three mismatches at position 3. Fraction of opened FQHs after 18 h of incubation with 1 nM of respective target DNA and with and without the addition of NPs. Unpaired t test results: * *p* > 0.05 and ** *p* < 0.05. Adapted with permission from Ochmann et al. [42] Copyright © 2019 American Chemical Society.

An optical nanoantenna was applied to the detection of Zika nucleic acids using a direct physical fluorescence amplification mechanism (Figure 8) [42]. Specifically, a DNA origami scaffold that incorporated noble metal nanoparticles was designed to fabricate a plasmonic hotspot. The modular structure of the DNA origami provides the possibility of placing a fluorescence-quenching hairpin (FQH) in the plasmonic hotspot, near a noble metal nanoparticle or in the gap between two nanoparticles that act as the optical antenna. The detection of Zika artificial DNA and RNA sequences with fluorescence enhancement was performed in either buffer or human blood serum. The assay sensitivity as well as the multiplexing capacity was assessed by examining single nucleotide variations using a fluorescence barcode in the origami base. The comparison between fluorescence scans from two different optical nanoantennas could be differentiated by the signal arising from green excitation. The two types of optical antennas were immobilized on the same surface and the signal was recorded after an incubation with both target DNAs overnight. Although the detection of DNA-spiked human serum samples and multiplexing analysis of different targets are remarkable advancements, the method proposed the optimization of the optical antenna by the introduction of multiplication processes for further enhancement of the fluorescence signal.

## 3. Single-Molecule Biosensing Strategies Based on Nanohole Arrays

Nanohole arrays are nanostructures fabricated on metallic materials that exhibit a high aspect-ratio and large electric field intensities in the subwavelength regime based on extraordinary optical transmission (EOT) [55]. The utilization of metallic surfaces with EOT implies that surface plasmons could be excited with an incoherent source of light. The spectral analysis of the transmission peaks generated by changes in the local refractive index can be monitored through a portable spectrometer using various plasmonic modes [56]. In comparison to other SPR arrangements, nanohole arrays enable several scattering orders that rely on the shape, size, periodicity, dielectric properties and composition of the materials [55]. These unique optical properties have contributed to improving the sensitivity of conventional SPR biosensors [33,43].

For example, the adsorption of immobilized molecules is described using DNA origami nanostructures as molecular probes and nanohole arrays as sensing mechanisms [39]. In particular, DNA origami triangles were captured on the exposed SiO_2_ bottom surface of gold nanoholes. The characterization of the adsorption process involved the investigation of several parameters including nanohole diameter, DNA origami concentrations and adsorption time. Experimental results showed that the buffer strength had a strong influence on DNA origami adsorption. Similarly, the utilization of low buffer strengths combined with low Mg^2+^ concentrations, low DNA origami concentrations, and long incubation times produced nanoholes occupied by only a single DNA origami triangle. However, the low surface mobility of the adsorbed DNA origami led to reduced localization accuracy inside the nanoholes, thus preventing their contact with the oxide surface. To overcome these limitations, the authors proposed the modification of the adsorption buffer and the employment of other substrate materials for the fabrication of the nanohole arrays.

**Figure 9:**
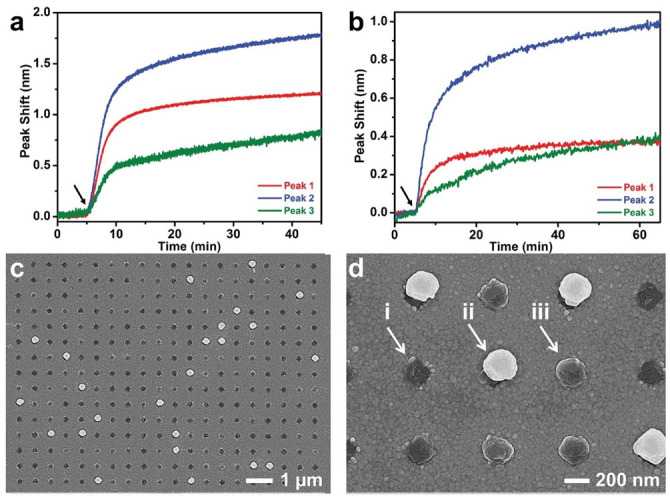
This is a figure. Selective adsorption of virus-like particles into functionalized nanoholes. Nanohole SPR experiments were performed on (**a**) nonfunctionalized and (**b**) mPEG-SH-functionalized nanohole arrays. Spectral shifts as a function of time were recorded for all three transmission peaks. The baseline signal corresponds to aqueous buffer solution. Then, 0.3 mg mL^−1^ virus-like particles were added (denoted by arrow) and the particle rupture process was tracked. SEM images obtained at (**c**) 20,000× and (**d**) 40,000× magnification demonstrate that single virus-like particles adsorbed into individual nanoholes on the mPEG-SH-functionalized nanohole array. In part (**d**), arrows indicate the three cases: (i) no particle in the nanohole; (ii) one particle partially inside the nanohole; and (iii) one particle predominately inside the nanohole. Aside from the nanohole positions, nonspecific adsorption of virus-like particles was not observed on the mPEG-SH-functionalized gold surface. Adapted with permission from Jackman et al. [43] Copyright © 2016 Wiley.

Jackman et al. designed a periodic nanohole array platform to capture single virus-like particles and virucidal drug candidates [43]. The selection of the suitable nanohole dimensions including diameter and height was calculated by nanoparticle tracking analysis of dengue virus-like particles before and after treatment with a virucidal drug candidate (Figure 9). The work specifically focused on the immobilization over gold nanoholes of lipid bilayer membranes consisting of cholesterol-enriched model virus particles. The low surface coverage of single virus particles was achieved due to the functionalization of nanoholes with thiol-terminated methoxy polyethylene(glycol) (mPEG-SH). The antifouling properties conferred by the mPEG-SH functionalized nanoholes ensured that vesicle adsorption only occurred inside the nanohole preventing the adsorption on the sidewalls and the top surface of gold nanoholes. The efficiency of virus-like particle adsorption within the nanoholes was evaluated by tracking changes in the wavelength position of transmission peaks. The increment of refractive index resulted in the shift of all three transmission peaks toward longer wavelengths. Further experiments verified with Scanning electron microscopy (SEM) images confirmed that 82% of the virus particles were mainly inside the nanohole. This strategy also enabled the specific and ultrasensitive detection of the virucidal peptide-induced particle rupture in the two nanohole configurations. The virucidal activity of α-helical (AH) peptide is capable of the rupture of lipid vesicles while promoting lipid bilayer formation on the sidewalls of the nonfunctionalized gold nanoholes. Therefore, monitoring of the transmission peaks served to detect virus-like particle adsorption and peptide-induced rupture on the more sensitive position of the nanohole. The work contributed to the characterization of single lipid layers according to the size of the deposited molecules. The investigation also concentrated on single particle detection and receptor–ligand interactions, however the study of virus particle conformation on the activity of virucidal drugs will help to improve the contribution of nanoplasmonic biosensing to the analysis of single particles.

A hybrid approach consisting of plasmonic silver nanohole arrays and second harmonic generation (SHG) microscopy imaging was used to visualize three-dimensional (3D) orientation of individual rhodamine 6G (R6G) molecules [44]. To demonstrate single-molecule detection, SHG and two-photon fluorescence microscopy experiments along with finite-difference time-domain (FDTD) simulations were conducted. The numerical simulation results indicated a ~10^6^-fold nonlinear enhancement of the electromagnetic field at the surface hot spots on the plasmonic nanohole substrate in comparison to R6G molecules immobilized on a glass. The comparison of experimental data with dipole emission images using the template matching algorithm revealed the position and 3D orientation of R6G molecules. Although this work mainly focused on the characterization of orientation imaging, it also provides the basis to improve the resolution of SHG signatures for single molecule detection of biological cells thanks to the integration of plasmonic silver nanohole structures.

## 4. Single-Molecule Biosensing Strategies Based on Plasmonic Nanoparticles

Metallic nanoparticles (e.g., nanorods, Ag NPs) offer important benefits for single-molecule biosensing due to their optical and electromagnetic properties at the nanoscale level [9]. Nanoparticle-based plasmonic biosensors usually exploit the unique scattering and absorption spectra of localized surface plasmon resonance (LSPR) [57]. In the case of gold and silver nanoparticles, localized surface plasmon occurs at visible-NIR wavelengths (400−100 nm), which is compatible with optical microscopy for subwavelength imaging and single molecule visualization [4].

**Figure 10:**
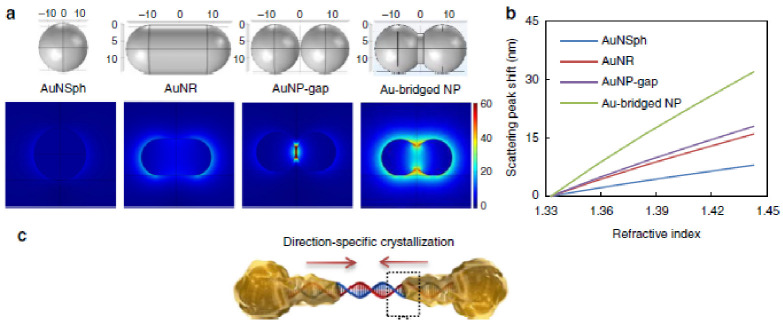
Synthesis-by-design of plasmonic nanoparticles (NPs) in solution. (**a**) Illustrations of the designed NP models (upper; dimensional unit, nm) and plasmon resonance electric field patterns (below; unit, Vm^−1^) generated by numerical simulations. (**b**) Linear fits to localized surface plasmon resonance (LSPR) wavelength shifts vs. changes in the refractive index (RI) of the NP surroundings. (**c**) Schematic diagram showing the early stage of direction-specific, contour-following, and shape-controlled crystallization of Au atoms by reducing AuCl4−with NH3OH^+^.The water interface of DNA provides precise controllability under the synthetic conditions at pH 5 and 4, generating NPs with nanobridges and nanogaps respectively. Adapted with permission from Ma et al. [58] Copyright © 2019 Nature.

The variety of dimensions, chemical composition, dielectric constants and boundary conditions can influence the collective oscillation modes of the conduction electrons in the particle, thereby determining the frequency of the plasmon resonance [4]. Owing to the electric field associated with the plasmon resonance, the interaction between individual molecules and metallic particles leads to frequency shifts on the plasmon resonance. Nanoparticle morphology can also affect the efficiency of plasmonic sensing for monitoring biomolecular interactions [8]. Therefore, the composition and shape of plasmonic nanoparticles are critical aspects for the design of single-molecule plasmonic sensors. In general, plasmonic nanoparticles based on sphere and rod geometries are commonly used in biomedical analysis due to their relatively narrow size distribution (Figure 10) [58].

Metallic nanoparticles require surface biofunctionalization to enable biomolecule detection. Because of their physical properties, noble metal nanostructures can be chemically activated through distinct procedures involving strategies from covalent thiol-chemistries to polyethylene glycol (PEG). Their versatility and biocompatibility also ensure that a broad range of biological receptors can be effectively immobilized on their surface. These particular features along with the large surface area-to-volume ratio make them perfectly suited for capturing particles at the single-molecule level.

This section provides an overview of technological advancements and applications of metallic nanoparticles for the analysis of single molecules using plasmonic biosensing schemes (see Table 2).

### 4.1. Single-Nucleic Acid Detection

**Figure 11:**
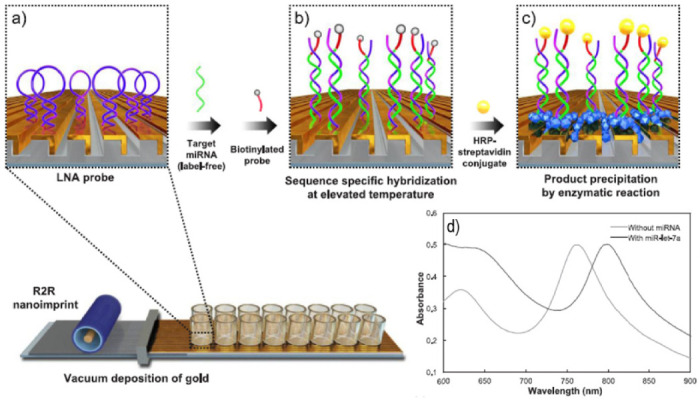
Schematic of the signal-amplified LSPR miRNA sensing platform on a scalable, flexible, transparent three-dimensional (3D) plasmonic nanostructure. (**a**) Self-assembled monolayer (SAM) formation with a hairpin LNA probe, (**b**) hybridization with miRNAs at elevated temperature followed by treatment with a biotinylated signaling probe, and (**c**) binding with HRP-streptavidin followed by enzymatic reaction converting soluble substrate to insoluble precipitate.; (**d**) LSPR spectra of patterns after incubation in the absence (**gray**) or presence of target miR-NA (**black**). Adapted with permission from Na et al. [59] Copyright © 2018 Elsevier.

Among nucleic acids, microRNAs (miRNAs) have become essential biomarkers in the diagnosis of pathological processes associated with cancer, diabetes or neurodegenerative diseases. Since miRNAs are noncoding RNAs short in length (19–22 nucleotides) and little abundant in biological fluids, detection of their expression levels could mainly be achieved by a real-time quantitative polymerase chain reaction (qRT-PCR) and other electrochemical or fluorescence-dependent methods. Recent label-free plasmonic sensing approaches have demonstrated their capacity for the quantitative determination of miRNA. Thus, Na et al. proposed a scalable and flexible LSPR platform capable of discriminating single-base mismatches in clinical samples [59]. The biosensing scheme involved the fabrication of 3D plasmonic nanostructures on gold strips wherein self-assembled monolayers (SAM) with a hairpin locked nucleic acid (LNA) probe were formed. The hybridization with miRNAs at different melting temperatures enable the determination of perfectly matched or single base mismatched miRNAs. The method also made use of a signal amplification procedure consisting of a treatment with a biotinylated signaling probe, followed by HRP-streptavidin binding and the enzymatic reaction that converted the soluble substrate to insoluble precipitate (Figure 11). The reaction resulted in a distinguishable LSPR peak shift allowing the detection of the target miRNA detection at attomole levels. Likewise, miRNA was also quantified in total RNA extracts from primary cancer cell lines showing high specificity with regard to structurally similar RNA molecules in biological samples. However, the main advantage of the proposed method over qRT-PCR is the possibility of discriminating single base mismatch without labeling, reverse transcription, or gene amplification. The plasmonic biosensor demonstrated the ability to distinguish miRNA nonspecific sequences containing only a single nucleotide substitution due to the denaturation of thermally unstable base pairing after incubation at elevated temperatures.

Single nucleotide mismatch detection has also been performed by an LSPR biosensor using thiolated single strand oligonucleotides immobilized on nanostructured LSPR sensors chips [60]. Two distinct functionalization strategies involving the utilization of gold nanodisks were evaluated. First, glass slides decorated with gold nanodisks provided optical signals corresponding to a single particle due to distribution and separation of nanostructures on the substrate, avoiding both far and near field coupling between the particles. The second type of sensor chips involved silicon oxide films (thickness 10 nm) deposited on top of gold nanodisks. Both Au and SiO_2_ sensor chips yielded LSPR signals because of the refractive index changes following the DNA hybridization processes. The immobilization of 5’-thiol modified 35-mer ssDNA probes onto the Au nanodisks array exhibited high efficiency displaying a surface coverage of 2.8 ± 0.1 × 10^12^/cm^2^. However, the work primarily focused on the investigation of the hybridization rate constants between surface-bound DNA probes and DNA target strands in solution. Particularly, a limit of detection of 10 nM and 13 nM was obtained when detecting perfect matching (PM) and mismatching (MM) targets for 93-mer sequences, respectively. The kinetic analysis and time dependence of two-component systems (PM and MM) were studied in comparison to a single-component system. Results showed that the MM strands fractions could be quantified in PM-MM binary solutions by monitoring the intensity of the relative LSPR signal at a fixed time. In addition, the hybridization kinetics displayed lower rates for MM with respect to PM target, whereas the competitive hybridization showed that MM sequences could kinetically hinder the hybridization rate of PM strands. These findings suggest that LSPR measurements could be used for detecting single nucleotide polymorphisms in a single step by means of the time response.

**Figure 12:**
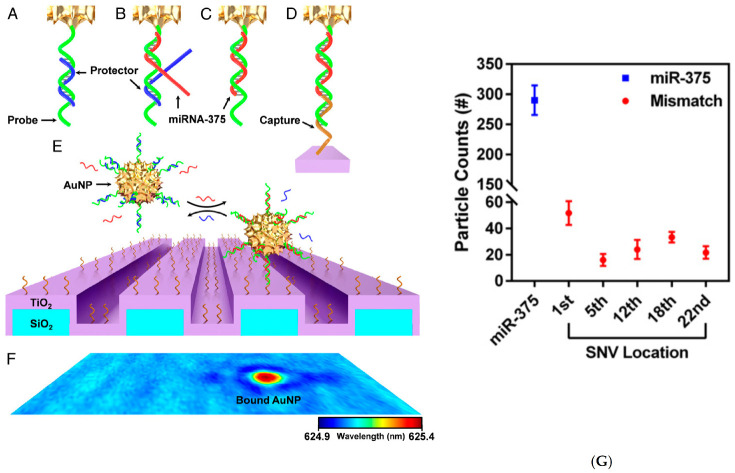
Schemes follow another format. If there are multiple panels, they should be listed as: (**A**) Components of the toehold DNA–AuNP and miR detection by PC biosensors. DNA hybridization probes are conjugated to 100-nm diameter AuNPs. (**A**) The gold-conjugated DNA probe (green) is bound by a partially complementary protector (blue) preventing binding to the PC sensor (purple/blue structure). (**B**–**D**) miR (red) binds at the probe toehold (**B**), resulting in strand displacement of the protector and exposing additional probe sequence (**C**), which (**D**) stabilizes probe binding to the PC capture DNA on the biosensor surface (**D**). The free energy of the activation reaction can be tuned by the protector (blue) stoichiometry, thus enhancing mismatch selectivity. Bound particles (**E**) can be measured by a shift in the PC resonance wavelength (**F**). All images are not to scale. The PRAM assay images the number of surface-captured particles over time (after miR addition); (**G**) AuNP count quantification of miR-375 and the SNV cases. Adapted with permission from Canady et al. [61] Copyright © 2019 PNAS.

The capability to discriminate single-base mismatches was also demonstrated by a hybrid plasmonic-photonic coupling approach consisting of DNA modified gold nanoparticles attached to photonic crystals (PC) [61]. The proposed biosensor provided the molecular recognition of individual miRNA targets (LSPR wavelength of ~625 nm) with digital resolution via so-called Photonic Resonator Absorption Microscopy (PRAM). The coupling between the resonant wavelength of the conjugated-gold nanoparticle and the PC nanostructure enabled the local quenching of the reflected light intensity from the PC due to the presence of individual nanoparticles (Figure 12). Dynamic PRAM imaging allowed the quantification of particle count as a function of miR-375 concentration at 2 h (prostate cancer biomarker). The selectivity of the assay for single-mismatch discrimination was investigated using five different single-nucleotide variants of miRNA-375. A remarkable decrease in particle count was observed for all five single nucleotide variants over time, with a range of ~83–94% signal reduction at 2 h. Therefore, through the synergistic coupling between the two resonators, digital detection of AuNPs served to selectively discriminate single-base mismatches, building the basis for direct diagnostics in complex background. 

A singular approach involving core−satellite plasmon rulers and dark-field imaging was used to study the dynamics of DNA/RNA displacement at the surface of gold nanoparticles [62]. The synthesis of core-satellite plasmon rulers implied the assembly of “satellite” gold nanoparticles to a “core” nanoparticle by duplex DNA. The separation of the satellite from the core due to the strand displacement induced a shift of scattering wavelength along with a change of color that was monitored by dark-field microscopy. The kinetics of toehold-mediated strand displacement was studied at the single-molecule level using microRNA-21 (miRNA-21) as invader strands. The calculation of reaction constants in cell lysates also allowed deducing the expression of miRNA-21 in living HeLa cells. Results were confirmed with a conventional fluorescence method, enabling the kinetics study at lower concentrations, even at the single-molecule level, with the proposed method in comparison to the fluorescence assay. Therefore, these findings suggest that the utilization of plasmon rulers in combination with microscopy imaging offers significant advances for single-molecule over fluorescence and sequencing methods. 

Another single-nucleotide mismatch distinguishing strategy was developed using a nanohybrid system based on LSPR-induced fluorescence [63]. The detection of nucleotide DNA sequences benefited from the formation of a sensing probe composed of gold nanoparticles functionalized with SiO_2_-quantum dots and subsequently conjugated to a molecular beacon. The biosensing mechanism relied specifically on the triggering of fluorescence changes in the quantum dots owing to the LSPR signal induced from gold nanoparticles upon hybridization of the targeted DNA. Although the method concentrated mostly on the characterization of the colloidal stability of gold nanoparticles, the efficiency of the probe for targeting non-complementary sequences was also demonstrated. Therefore, the plasmon-enhanced fluorescence effect of AuNPs on SiO2-Quantum dots upon binding offered either the opportunity to detect perfect complementary nucleotide sequences at 10 fg/mL or single-base nucleotide mismatch and non-complementary sequence target.

### 4.2. Single-Protein Interaction Detection

The quantification of proteins at ultra-low concentrations is of paramount importance for the diagnosis of diseases at an early stage. In this regard, plasmonic nanoparticles are perfectly suited not only for the detection of single protein at molecular level but also for the investigation of the interaction between proteins and their specific recognition element.

**Figure 13:**
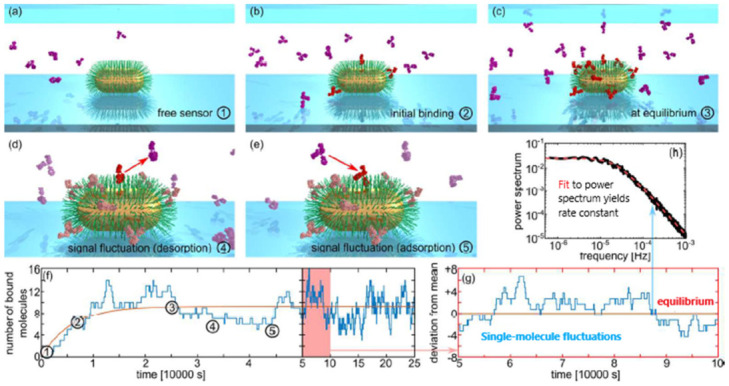
(**a**–**e**) Nanoplasmonic LSPR sensing of single-molecule equilibrium fluctuations. (**f**) Simulated response of a nanosensor to binding of analyte molecules (blue) and the corresponding average over many sensors (orange). ① A nanorod covered with a receptor layer is exposed to flow of analyte solution. ② Analyte molecules bind to the receptors and surface coverage eventually reaches equilibrium ③, at which time averaged numbers of associating and dissociating molecules are equal. However, molecules continue to ④ dissociate and ⑤ associate at random times. This causes short time-scale fluctuations of the surface occupancy around the mean. (**g**) Once equilibrium is reached, molecules associate and dissociate stochastically with rates determined by k_on_, k_off_, and ρ, causing single-molecule fluctuations around the equilibrium signal. In this regime, the system ceases to be mass transport limited. (**h**) Analysis of power spectrum of equilibrium fluctuations yields accurate values of rate constants. Adapted with permission from Aćimović et al. [64] Copyright © 2018 American Chemical Society.

Monitoring single-molecule binding events is of particular interest for understanding the dynamics of antibody-antigen interactions in the immune-related response. In this sense, LSPR biosensing offers the possibility of tracking spectral shifts over time enabling the study of antibody-antigen kinetics such as association constants, equilibrium fluctuations or denaturation dynamics of large proteins [64]. Particularly, a LSPR based-approach investigated the interaction dynamics between PEG-specific immunoglobulin antibody (Anti-PEG) and surface-thiolated polyethylene glycol (PEG) brushes coated on gold nanorods. The device made use of an opto-mechanical set-up comprising a standard inverted microscope with a CMOS camera and an imaging spectrometer for LSPR detection. Analysis of PEG/Anti-PEG interaction kinetics revealed a strong association between Anti-PEG concentrations and surface coverage, showing a hierarchical-like evolution trend (Figure 13). The quantification of single-molecule equilibrium fluctuations required the collection of LSPR data at the single nanoparticle level during periods of time of more than 24 h, much longer than previous experiments based on short time scales. This special feature allowed the calculation of association rates as a function of the arrival and binding times of molecules to the sensor surface. The possibility of using different set-ups whenever the amplitude of white noise (obtained before calculating the spectra) was larger than the single-molecule signal confirmed the flexibility of the optofluidic system. Similarly, the capability for analyzing not only interaction kinetics but also other dynamic processes involved in binding events such as surface diffusion or conformational changes are other potential advantages of this plasmonic nanoparticle biosensor.

Another plasmonic application utilized gold nanorods for investigating the kinetics and binding position of protein biomarkers [65]. In particular, this method proposed the calculation of ideal detection volumes for single molecules. Simulation results showed that single molecule analysis required sufficiently small volumes (~990 nm^3^) to allow the detection of protein molecules (~5–10 nm.) while ensuring the suppression of the background noise. To improve the assay sensitivity, the effect of the environment including buffer, substrate and type of ligand was also studied using streptavidin-R-phycoerythrin molecules. Whereas the substrate and buffer could drastically affect the sensitivity, the ligand diameter did not influence the assay performance. Nevertheless, the sensitivity was dependent on the length of the ligand since it determines the distance of the biomolecule from the gold nanorod. Finally, the detection of single molecules was further enhanced using a dielectric cladding layer around the nanorod except the two tips. This strategy pursued that the biomolecules were only attached to the tips, where the plasmonic field enhancement is at its maximum (hot spots). By taking advantage of the intense electric field confinement around tips, the sensitivity increased four orders of magnitude when monitoring thyroglobulin and glycoprotein biomarkers. Thus, LSPR biosensors based on the attachment of molecules on the tips of gold nanorods demonstrated higher sensitivities for label-free detection of single protein molecules in comparison with other optical sensing approaches.

### 4.3. Single-Cell Detection

A biosensor application involving plasmon-enhanced fluorescence has been used for single-molecule fluorescence imaging of Vibrio cholerae living cells [66]. The work demonstrated the enhancement of single-molecule fluorescence intensity due to the coupling of living cells to gold nanotriangle arrays. Since this enhancement depends on the nanoparticles’ properties and dimensions, the plasmonic coupling was studied as a function of the surface coverage and LSPR frequencies of gold nanoparticles. As a result, nanotriangle arrays fabricated by nanosphere lithography on glass coverslips were characterized according to their morphology and diameter before being used as substrates for the imaging of living Vibrio cholerae cells. The pathogenicity of Vibrio cholerae relies on the expression of photoactivatable fluorescent proteins. Therefore, by monitoring V. cholerae cells on top of extracellular nanoparticles, single-molecule fluorescence is further improved due to the proteins (TcpP-PAmCherry) being positioned within the enhanced near field of the plasmonic nanotriangle arrays. In this localization, the fluorescence excitation and emission maxima wavelengths of TcpP-PAmCherry overlap well with the NTA LSPR frequencies. The optimization of the nanoparticle geometry and coverage led to a two-fold single-molecule fluorescence enhancement in live cells, improving the resolution of standard fluorescence single-molecule imaging methods.

Single-molecule sensitivity has also been proved using plasmon rulers for live-cell detection of secreted single protein molecules [67]. Plasmon rulers comprised dimers of identical gold nanoparticles linked by a single-aptamer that enabled the detection of single-molecule activity. In contrast to fluorescence-based single-molecule methods, plasmon rulers are capable of binding individual molecules without photobleaching or blinking. Their sensing mechanism relies on the generation of a light scattering spectrum when the surface plasmon resonance of the two gold nanoparticles couple due to their proximity. Therefore, the binding of target molecules to the aptamer originates conformational changes that induce the variation of interparticle distance and an observable change in the light scattering spectrum. In this work, a matrix metalloproteinase (MMP3) was selected as the target molecule because of its role in the dynamics involved in biochemical pathways in cancer. The kinetics of the binding between MMP3 proteins and the single aptamer of plasmon rulers were measured at the single-molecule level. It was observed that the binding of the MMP3 molecule to the aptamer between the two Au nanoparticles caused a decrease in the interparticle distance resulting in a spectral redshift. The reversibility of the aptamer-protein binding reaction represents an additional advantage over plasmon rulers that rely on the cleavage of their biomolecule substrate to generate a change in their interparticle distance. Therefore, the dynamic response of these plasmon rulers demonstrated their capability to measure single secreted molecules with high specificity and in a reversible manner.

## 5. Conclusions and Future Outlook

This work compiles recent progress in plasmonic applications for single-molecule biomedical analysis. The utilization of hybrid coupling technologies (i.e., electrical, fluorescence or microscopy) has opened the way toward the development of plasmonic ultrasensitive detection methods expected to be applied in clinical settings. Although label-free plasmonic sensing has been extensively exploited in medical research, the role of single molecule-based plasmonic platforms as diagnosis tools in the medical field is still at an early stage.

To overcome this limitation, current emerging technologies have prompted the fabrication of new plasmonic structures aimed at addressing the peculiarities of targeting individual molecules. Among them, the complementarity of plasmonic nanomaterials and nanopores has enabled the monitoring of single binding events with high precision. The development of plasmonic nanopores has benefited from the extraordinary optical properties of plasmonic surfaces and the molecular-scale dimensions of nanopores. Plasmonic nanopores have contributed to improving the dwelling times of analytes while facilitating molecule trapping into the active detection sites due to their enhanced field localization and thermophoretic capacity. Several meaningful works have attained single-molecule sensing of nucleic acids, proteins and pathogenic agents using plasmonic nanopores. However, the number of applications is still primarily limited to proof-of-concept demonstrations and optical characterizations supported by theoretical simulations. To date, most approaches conducted to investigate DNA sequencing and protein binding interactions do not exceed the requirements of commercially available instruments. Nonetheless, the possibility of fabricating nanopores integrated into plasmonic-nanofluidic platforms can lead to the multiplexed analysis of biomolecules with superior spectral resolution.

The other plasmonic strategy commonly applied to single-molecule biosensing involves the utilization of nanoparticles. Plasmonic nanoparticles offer the opportunity of providing higher sensitivities in comparison with two dimensional metallic films because of the enhancement of surface-to-volume ratios. The efficiency of plasmonic nanoparticle-based applications also relies on both the manipulation of these strongly-enhanced near fields and the engineering of nanoparticles assemblies. Although plasmonic biosensors have been mostly employed for targeting single-base DNA mismatches, single-cell biosensing also provides promising advantages thanks to the incorporation of far-field optical microscopy and fluorescent techniques. By integrating additional readers, single-molecule biosensing has been effectively applied to monitoring the dynamics of biological processes. In particular, the investigation of conformational changes and binding kinetics of individual molecules has been reported in various protein detection approaches.

In spite of these significant innovations, the state-of-the-art of single-molecule plasmonic biosensors is far from the clinical implementation as point-of-care devices. First, the fabrication of operational devices ready for personalized medicine requires the restriction of mass transport effects. The integration of nanofluidics into scalable platforms is essential for controlling sample volumes down to the subpicoliter range. Therefore, detection of single molecules involves the downscaling of fluidics to the dimensions of nanoplasmonic structures. In this sense, the selection of materials with specific geometries such as well-shaped nanoapertures enables the maximization of plasmon confinement and resonance intensity while leading to a substantial gaining of signal-to-background ratios. Similarly, further enhancement of detection rates and nanoscale volumes could help to design plasmonic sensors by fabricating arrays of nanoholes. Overall, building scaling nanosensors for biomolecular analysis of clinical samples demands high sensitivity and specificity. Since the presence of analytes in complex media can be outnumbered by non-target species, plasmonic sensors should exhibit high precision when quantifying biomolecular targets at single molecule levels. By addressing this gap, the number of biosensing applications in the clinical field could increase significantly. The development of plasmonic sensing systems with higher spatiotemporal resolutions will contribute to broadening the spectrum of single-molecule applications to other analytes such as exosomes or living cells. Furthermore, the search for nanomaterials with extended physicochemical stability and biocompatibility will refine the robustness of plasmonic configurations and support the progress towards functional devices at the point of need.

## Figures and Tables

**Table 1:** Key analytical features of plasmonic applications based on nanopores and nanohole arrays sensors classified according to the characteristics of target analyte, instrument configuration (namely sensing scheme or biological receptor) and detection format.

Target Analyte	Instrument Configuration	Detection Strategy	Reference
Nucleic acids	Nanopore-nanowells with fluorescence enhancement	Double-stranded single DNA molecules adsorption	[34]
	Graphene quantum dots with a nanopore (density functional theory calculations.)	DNA-graphene interactions and nucleobase (adenine, thymine, cytosine, and guanine) rotation	[35]
	Nanopore, bowtie, and bowtie-nanopore structures and SPR materials (chromium, aluminum, rhodium and graphene)	DNA nucleotides shifts in the SPR spectra through Bowtie-nanopore structures	[36]
	Two elongated nanodiscs in a gold bowtie nanoantenna	Single-DNA molecules in the nanopore gap monitoring (l intensity of light backscattered from the antenna)	[37]
	Inverted-bowtie gold plasmonic nanopore	DNA-protein interactions and DNA translocation (optical signals under different illumination conditions)	[38]
	Nanohole arrays	Single-DNA origami triangles were captured on SiO_2_ bottom surface of gold nanoholes	[39]
Proteins	Plasmonic nanopore tri-color fluorescence simulation and pattern-recognition algorithms	Individual protein translocations, human plasma proteome and cytokine recognition	[40]
	Plasmonic nanopore acting as nano tweezer	Individual beta-amylase proteins optical trapping	[41]
Infectious agents	Optical nanoantenna with direct physical fluorescence amplification	Single nucleotide variations in Zika artificial DNA origami buffer and human serum	[42]
	Nanohole array	Single dengue virus-like particles and virucidal drug candidates (spectral shifts of transmission peaks)	[43]
Organic compounds	Silver nanohole arrays and microscopy imaging	Individual rhodamine 6G (R6G) three-dimensional orientation	[44]

**Table 2:** Key analytical features of plasmonic nanoparticle-based sensors classified according to the characteristics of target analyte, instrument configuration (namely sensing scheme or biological receptor) and detection format.

Target Analyte	Instrument Configuration	Detection Format	Reference
Nucleic acids	LSPR platform with 3D plasmonic nanostructures fabricated on gold strips	Single base mismatched detection of microRNAs molecules at different melting temperatures	[59]
	LSPR platform with silicon oxide deposited on top of gold nanodisks	Single nucleotide perfect matching and mismatching	[60]
	Gold nanoparticles attached to photonic crystals (Photonic Resonator Absorption Microscopy)	Single-base mismatches of5 different single-nucleotide variants of microRNA-375	[61]
	Core−satellite plasmon rulers (gold nanoparticles) and dark-field microscopy imaging	microRNA-21 expression in living HeLa cells at single molecule level	[62]
	LSPR-induced fluorescence (of gold nanoparticles-SiO_2_-quantum dots conjugated to a molecular beacon)	Single-base nucleotide DNA mismatch	[63]
Proteins	LSPR platform with gold nanorods (CMOS camera and an imaging spectrometer)	Single-molecule equilibrium fluctuations (PEG/anti-PEG interactions)	[64]
	LSPR platform with gold nanorods	Kinetics and binding position of single thyroglobulin and glycoprotein molecules	[65]
Cells	LSPR with Nanotriangle arrays and fluorescence imaging	Single-molecule fluorescence detection of photoactivable *Vibrio cholerae* living cells	[66]
	Plasmon rulers linked by a single-aptamer	Binding of cell-secreted MMP3 proteins to aptamer at the single-molecule level	[67]
